# Coding and Classification Errors in Study of Psilocybin Services

**DOI:** 10.1001/jamanetworkopen.2026.44571

**Published:** 2026-10-16

The authors of the Original Investigation, “Safety and Mental Health Outcomes of Oregon State-Regulated Psilocybin Services,”^1^ published on August 19, 2026, in *JAMA Network Open*, have reported errors in the published article, including a coding error that misclassified reasons for seeking services and a small number of participants with miscategorized “other” race and 2 fewer participants with lifetime use of psilocybin than originally reported. The authors have explained what happened and the corrections needed to the text of the article, Tables, and Supplement in a Comment posted with the article.^2^ The article has been corrected.^1^
